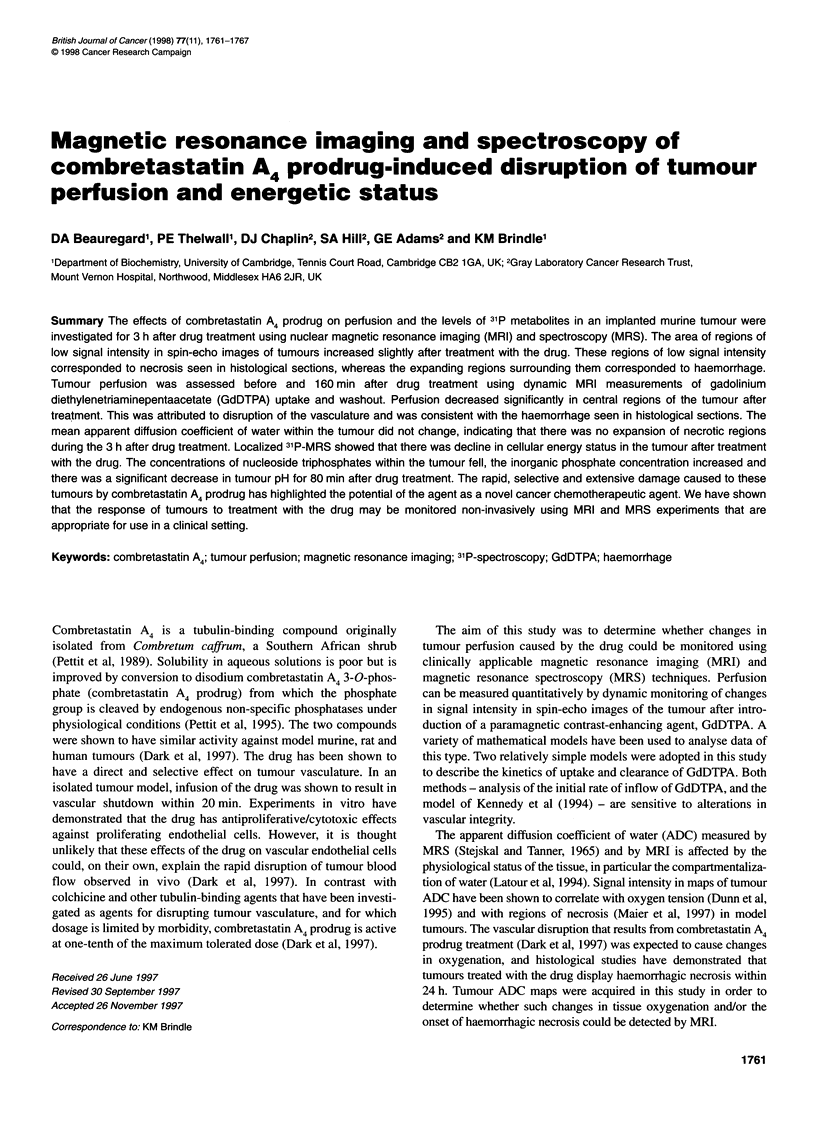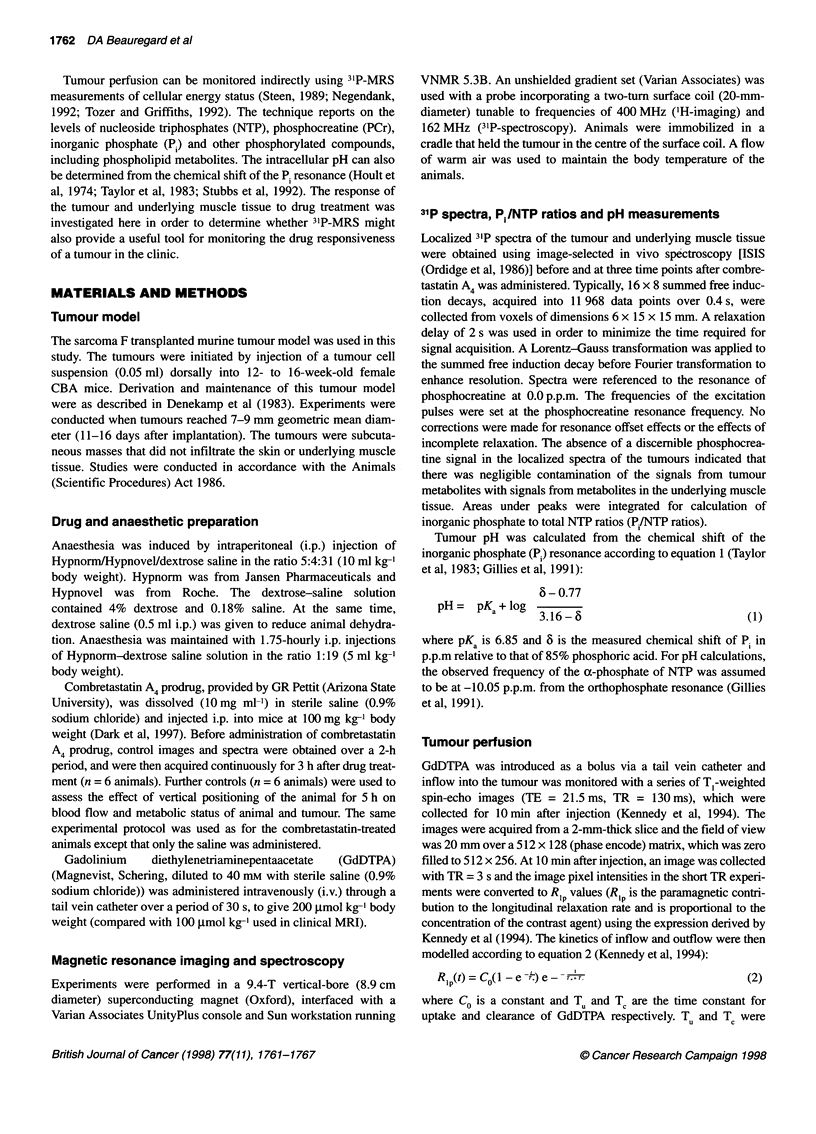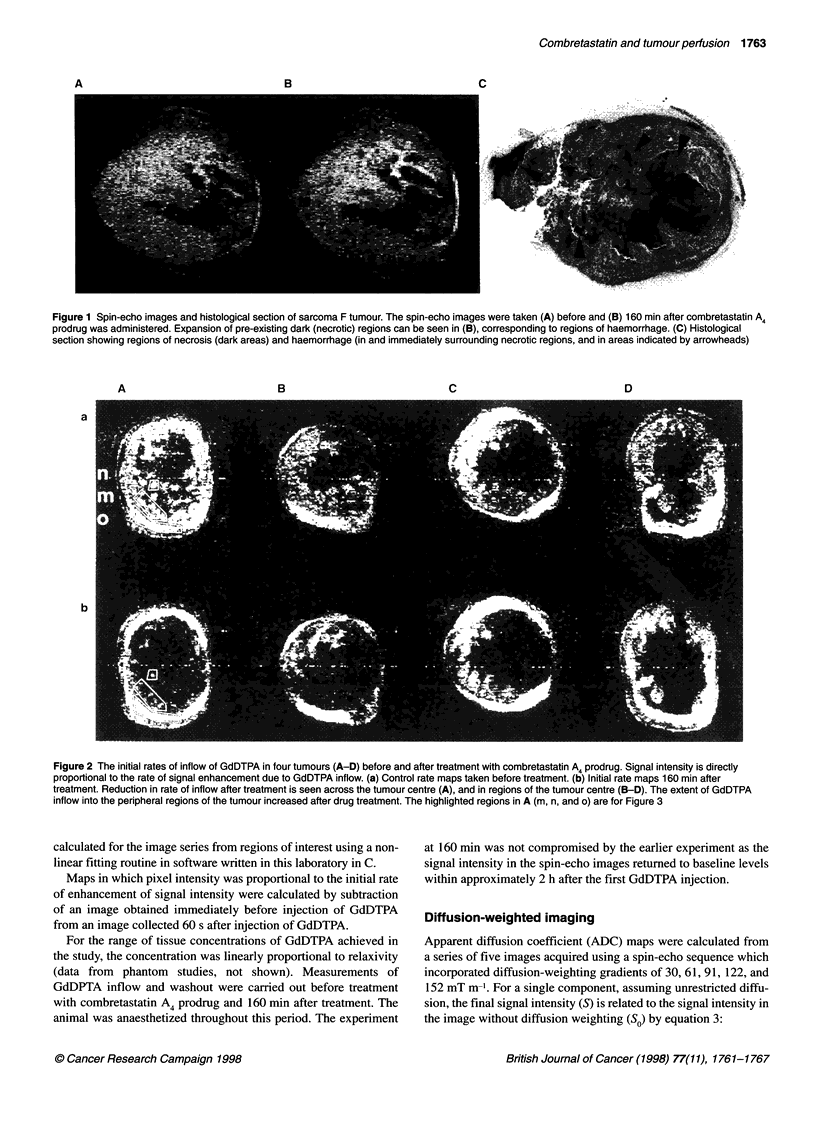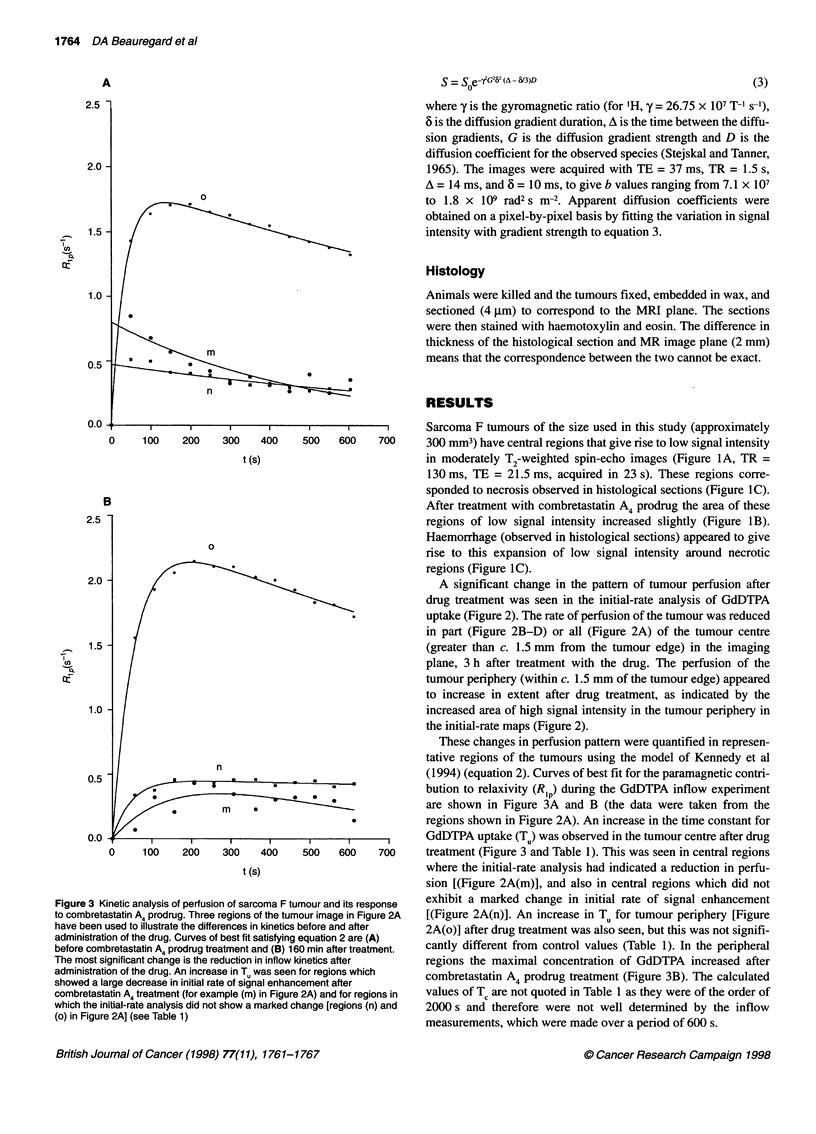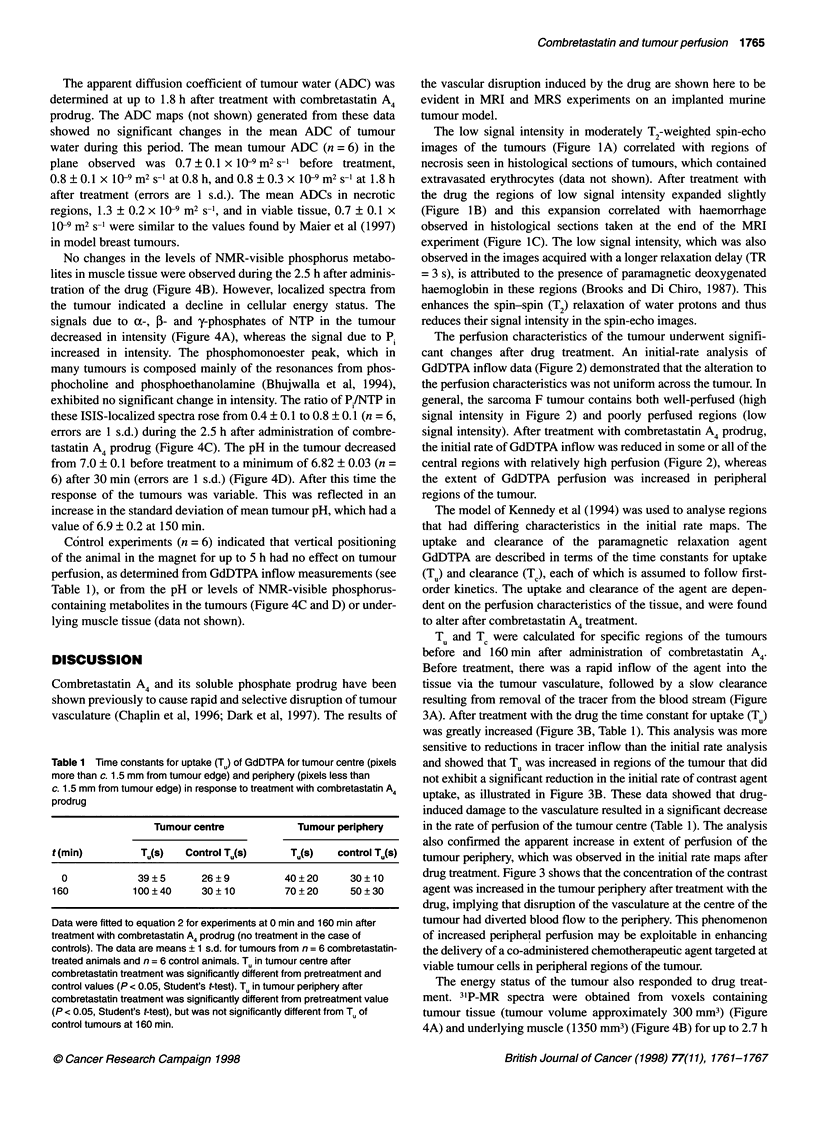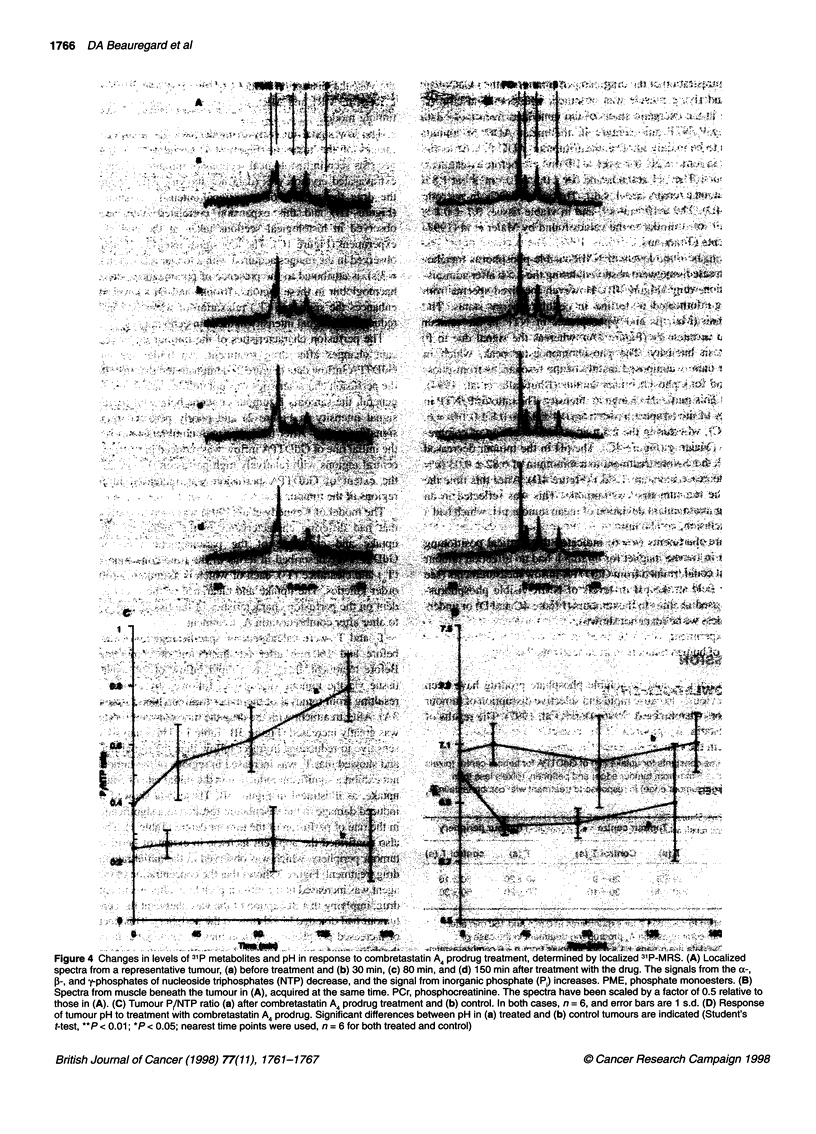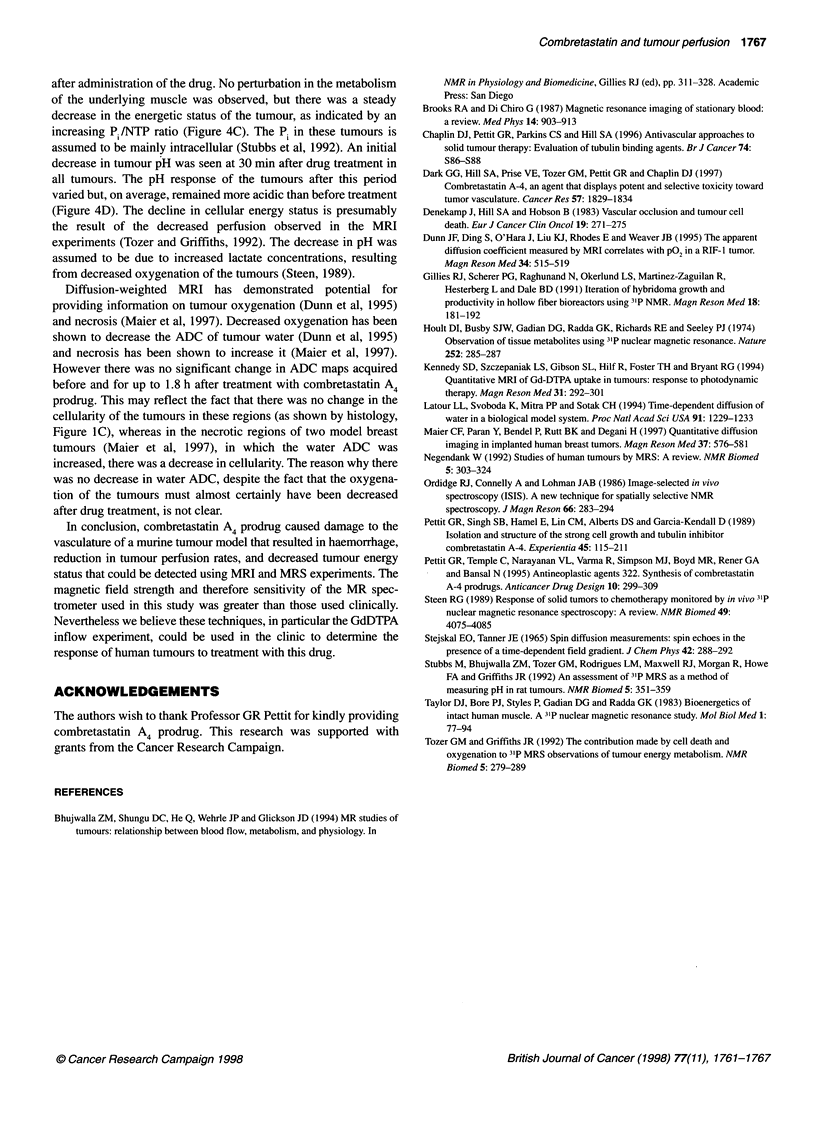# Magnetic resonance imaging and spectroscopy of combretastatin A4 prodrug-induced disruption of tumour perfusion and energetic status.

**DOI:** 10.1038/bjc.1998.294

**Published:** 1998-06

**Authors:** D. A. Beauregard, P. E. Thelwall, D. J. Chaplin, S. A. Hill, G. E. Adams, K. M. Brindle

**Affiliations:** Department of Biochemistry, University of Cambridge, UK.

## Abstract

**Images:**


*British Journal of Cancer* (1998) **77**(11), 1761–1767
© 1998 Cancer Research Campaign

### Magnetic resonance imaging and spectroscopy of combretastatin $A_4$ prodrug-induced disruption of tumour perfusion and energetic status

**DA Beauregard[1], PE Thelwall[1], DJ Chaplin[2], SA Hill[2], GE Adams[2] and KM Brindle[1]**

[1]Department of Biochemistry, University of Cambridge, Tennis Court Road, Cambridge CB2 1GA, UK; [2]Gray Laboratory Cancer Research Trust, Mount Vernon Hospital, Northwood, Middlesex HA6 2JR, UK

**Summary** The effects of combretastatin $A_4$ prodrug on perfusion and the levels of $^{31}P$ metabolites in an implanted murine tumour were investigated for 3 h after drug treatment using nuclear magnetic resonance imaging (MRI) and spectroscopy (MRS). The area of regions of low signal intensity in spin-echo images of tumours increased slightly after treatment with the drug. These regions of low signal intensity corresponded to necrosis seen in histological sections, whereas the expanding regions surrounding them corresponded to haemorrhage. Tumour perfusion was assessed before and 160 min after drug treatment using dynamic MRI measurements of gadolinium diethylenetriaminepentaacetate (GdDTPA) uptake and washout. Perfusion decreased significantly in central regions of the tumour after treatment. This was attributed to disruption of the vasculature and was consistent with the haemorrhage seen in histological sections. The mean apparent diffusion coefficient of water within the tumour did not change, indicating that there was no expansion of necrotic regions during the 3 h after drug treatment. Localized $^{31}P$-MRS showed that there was decline in cellular energy status in the tumour after treatment with the drug. The concentrations of nucleoside triphosphates within the tumour fell, the inorganic phosphate concentration increased and there was a significant decrease in tumour pH for 80 min after drug treatment. The rapid, selective and extensive damage caused to these tumours by combretastatin $A_4$ prodrug has highlighted the potential of the agent as a novel cancer chemotherapeutic agent. We have shown that the response of tumours to treatment with the drug may be monitored non-invasively using MRI and MRS experiments that are appropriate for use in a clinical setting.

**Keywords:** combretastatin $A_4$; tumour perfusion; magnetic resonance imaging; $^{31}P$-spectroscopy; GdDTPA; haemorrhage

Combretastatin $A_4$ is a tubulin-binding compound originally isolated from *Combretum caffrum*, a Southern African shrub (Pettit et al, 1989). Solubility in aqueous solutions is poor but is improved by conversion to disodium combretastatin $A_4$ 3-*O*-phosphate (combretastatin $A_4$ prodrug) from which the phosphate group is cleaved by endogenous non-specific phosphatases under physiological conditions (Pettit et al, 1995). The two compounds were shown to have similar activity against model murine, rat and human tumours (Dark et al, 1997). The drug has been shown to have a direct and selective effect on tumour vasculature. In an isolated tumour model, infusion of the drug was shown to result in vascular shutdown within 20 min. Experiments in vitro have demonstrated that the drug has antiproliferative/cytotoxic effects against proliferating endothelial cells. However, it is thought unlikely that these effects of the drug on vascular endothelial cells could, on their own, explain the rapid disruption of tumour blood flow observed in vivo (Dark et al, 1997). In contrast with colchicine and other tubulin-binding agents that have been investigated as agents for disrupting tumour vasculature, and for which dosage is limited by morbidity, combretastatin $A_4$ prodrug is active at one-tenth of the maximum tolerated dose (Dark et al, 1997).

*Received 26 June 1997*
*Revised 30 September 1997*
*Accepted 26 November 1997*

*Correspondence to:* KM Brindle

The aim of this study was to determine whether changes in tumour perfusion caused by the drug could be monitored using clinically applicable magnetic resonance imaging (MRI) and magnetic resonance spectroscopy (MRS) techniques. Perfusion can be measured quantitatively by dynamic monitoring of changes in signal intensity in spin-echo images of the tumour after introduction of a paramagnetic contrast-enhancing agent, GdDTPA. A variety of mathematical models have been used to analyse data of this type. Two relatively simple models were adopted in this study to describe the kinetics of uptake and clearance of GdDTPA. Both methods – analysis of the initial rate of inflow of GdDTPA, and the model of Kennedy et al (1994) – are sensitive to alterations in vascular integrity.

The apparent diffusion coefficient of water (ADC) measured by MRS (Stejskal and Tanner, 1965) and by MRI is affected by the physiological status of the tissue, in particular the compartmentalization of water (Latour et al, 1994). Signal intensity in maps of tumour ADC have been shown to correlate with oxygen tension (Dunn et al, 1995) and with regions of necrosis (Maier et al, 1997) in model tumours. The vascular disruption that results from combretastatin $A_4$ prodrug treatment (Dark et al, 1997) was expected to cause changes in oxygenation, and histological studies have demonstrated that tumours treated with the drug display haemorrhagic necrosis within 24 h. Tumour ADC maps were acquired in this study in order to determine whether such changes in tissue oxygenation and/or the onset of haemorrhagic necrosis could be detected by MRI.

Tumour perfusion can be monitored indirectly using $^{31}$P-MRS measurements of cellular energy status (Steen, 1989; Negendank, 1992; Tozer and Griffiths, 1992). The technique reports on the levels of nucleoside triphosphates (NTP), phosphocreatine (PCr), inorganic phosphate ($P_i$) and other phosphorylated compounds, including phospholipid metabolites. The intracellular pH can also be determined from the chemical shift of the $P_i$ resonance (Hoult et al, 1974; Taylor et al, 1983; Stubbs et al, 1992). The response of the tumour and underlying muscle tissue to drug treatment was investigated here in order to determine whether $^{31}$P-MRS might also provide a useful tool for monitoring the drug responsiveness of a tumour in the clinic.

### MATERIALS AND METHODS

### Tumour model

The sarcoma F transplanted murine tumour model was used in this study. The tumours were initiated by injection of a tumour cell suspension (0.05 ml) dorsally into 12- to 16-week-old female CBA mice. Derivation and maintenance of this tumour model were as described in Denekamp et al (1983). Experiments were conducted when tumours reached 7–9 mm geometric mean diameter (11–16 days after implantation). The tumours were subcutaneous masses that did not infiltrate the skin or underlying muscle tissue. Studies were conducted in accordance with the Animals (Scientific Procedures) Act 1986.

### Drug and anaesthetic preparation

Anaesthesia was induced by intraperitoneal (i.p.) injection of Hypnorm/Hypnovel/dextrose saline in the ratio 5:4:31 (10 ml kg$^{-1}$ body weight). Hypnorm was from Jansen Pharmaceuticals and Hypnovel was from Roche. The dextrose–saline solution contained 4% dextrose and 0.18% saline. At the same time, dextrose saline (0.5 ml i.p.) was given to reduce animal dehydration. Anaesthesia was maintained with 1.75-hourly i.p. injections of Hypnorm–dextrose saline solution in the ratio 1:19 (5 ml kg$^{-1}$ body weight).

Combretastatin $A_4$ prodrug, provided by GR Pettit (Arizona State University), was dissolved (10 mg ml$^{-1}$) in sterile saline (0.9% sodium chloride) and injected i.p. into mice at 100 mg kg$^{-1}$ body weight (Dark et al, 1997). Before administration of combretastatin $A_4$ prodrug, control images and spectra were obtained over a 2-h period, and were then acquired continuously for 3 h after drug treatment ($n = 6$ animals). Further controls ($n = 6$ animals) were used to assess the effect of vertical positioning of the animal for 5 h on blood flow and metabolic status of animal and tumour. The same experimental protocol was used as for the combretastatin-treated animals except that only the saline was administered.

Gadolinium diethylenetriaminepentaacetate (GdDTPA) (Magnevist, Schering, diluted to 40 mM with sterile saline (0.9% sodium chloride)) was administered intravenously (i.v.) through a tail vein catheter over a period of 30 s, to give 200 μmol kg$^{-1}$ body weight (compared with 100 μmol kg$^{-1}$ used in clinical MRI).

### Magnetic resonance imaging and spectroscopy

Experiments were performed in a 9.4-T vertical-bore (8.9 cm diameter) superconducting magnet (Oxford), interfaced with a Varian Associates UnityPlus console and Sun workstation running VNMR 5.3B. An unshielded gradient set (Varian Associates) was used with a probe incorporating a two-turn surface coil (20-mm-diameter) tunable to frequencies of 400 MHz ($^1$H-imaging) and 162 MHz ($^{31}$P-spectroscopy). Animals were immobilized in a cradle that held the tumour in the centre of the surface coil. A flow of warm air was used to maintain the body temperature of the animals.

### $^{31}$P spectra, $P_i$/NTP ratios and pH measurements

Localized $^{31}$P spectra of the tumour and underlying muscle tissue were obtained using image-selected in vivo spectroscopy [ISIS (Ordidge et al, 1986)] before and at three time points after combretastatin $A_4$ was administered. Typically, 16 × 8 summed free induction decays, acquired into 11 968 data points over 0.4 s, were collected from voxels of dimensions 6 × 15 × 15 mm. A relaxation delay of 2 s was used in order to minimize the time required for signal acquisition. A Lorentz–Gauss transformation was applied to the summed free induction decay before Fourier transformation to enhance resolution. Spectra were referenced to the resonance of phosphocreatine at 0.0 p.p.m. The frequencies of the excitation pulses were set at the phosphocreatine resonance frequency. No corrections were made for resonance offset effects or the effects of incomplete relaxation. The absence of a discernible phosphocreatine signal in the localized spectra of the tumours indicated that there was negligible contamination of the signals from tumour metabolites with signals from metabolites in the underlying muscle tissue. Areas under peaks were integrated for calculation of inorganic phosphate to total NTP ratios ($P_i$/NTP ratios).

Tumour pH was calculated from the chemical shift of the inorganic phosphate ($P_i$) resonance according to equation 1 (Taylor et al, 1983; Gillies et al, 1991):

$$\text{pH} = \text{p}K_a + \log \frac{\delta - 0.77}{3.16 - \delta} \quad (1)$$

where $pK_a$ is 6.85 and δ is the measured chemical shift of $P_i$ in p.p.m relative to that of 85% phosphoric acid. For pH calculations, the observed frequency of the α-phosphate of NTP was assumed to be at –10.05 p.p.m. from the orthophosphate resonance (Gillies et al, 1991).

### Tumour perfusion

GdDTPA was introduced as a bolus via a tail vein catheter and inflow into the tumour was monitored with a series of $T_1$-weighted spin-echo images (TE = 21.5 ms, TR = 130 ms), which were collected for 10 min after injection (Kennedy et al, 1994). The images were acquired from a 2-mm-thick slice and the field of view was 20 mm over a 512 × 128 (phase encode) matrix, which was zero filled to 512 × 256. At 10 min after injection, an image was collected with TR = 3 s and the image pixel intensities in the short TR experiments were converted to $R_{1p}$ values ($R_{1p}$ is the paramagnetic contribution to the longitudinal relaxation rate and is proportional to the concentration of the contrast agent) using the expression derived by Kennedy et al (1994). The kinetics of inflow and outflow were then modelled according to equation 2 (Kennedy et al, 1994):

$$R_{1p}(t) = C_0(1 - e^{-\frac{t}{T_u}})\, e - ^{-\frac{1}{T_u + T_c}} \quad (2)$$

where $C_0$ is a constant and $T_u$ and $T_c$ are the time constant for uptake and clearance of GdDTPA respectively. $T_u$ and $T_c$ were

*© Cancer Research Campaign 1998*

**Figure 1** Spin-echo images and histological section of sarcoma F tumour. The spin-echo images were taken (**A**) before and (**B**) 160 min after combretastatin $A_4$ prodrug was administered. Expansion of pre-existing dark (necrotic) regions can be seen in (**B**), corresponding to regions of haemorrhage. (**C**) Histological section showing regions of necrosis (dark areas) and haemorrhage (in and immediately surrounding necrotic regions, and in areas indicated by arrowheads)

**Figure 2** The initial rates of inflow of GdDTPA in four tumours (**A–D**) before and after treatment with combretastatin $A_4$ prodrug. Signal intensity is directly proportional to the rate of signal enhancement due to GdDTPA inflow. (**a**) Control rate maps taken before treatment. (**b**) Initial rate maps 160 min after treatment. Reduction in rate of inflow after treatment is seen across the tumour centre (**A**), and in regions of the tumour centre (**B–D**). The extent of GdDTPA inflow into the peripheral regions of the tumour increased after drug treatment. The highlighted regions in **A** (m, n, and o) are for Figure 3

calculated for the image series from regions of interest using a non-linear fitting routine in software written in this laboratory in C.

Maps in which pixel intensity was proportional to the initial rate of enhancement of signal intensity were calculated by subtraction of an image obtained immediately before injection of GdDTPA from an image collected 60 s after injection of GdDTPA.

For the range of tissue concentrations of GdDTPA achieved in the study, the concentration was linearly proportional to relaxivity (data from phantom studies, not shown). Measurements of GdDPTA inflow and washout were carried out before treatment with combretastatin $A_4$ prodrug and 160 min after treatment. The animal was anaesthetized throughout this period. The experiment at 160 min was not compromised by the earlier experiment as the signal intensity in the spin-echo images returned to baseline levels within approximately 2 h after the first GdDTPA injection.

### Diffusion-weighted imaging

Apparent diffusion coefficient (ADC) maps were calculated from a series of five images acquired using a spin-echo sequence which incorporated diffusion-weighting gradients of 30, 61, 91, 122, and 152 mT $m^{-1}$. For a single component, assuming unrestricted diffusion, the final signal intensity ($S$) is related to the signal intensity in the image without diffusion weighting ($S_0$) by equation 3:

**Figure 3** Kinetic analysis of perfusion of sarcoma F tumour and its response to combretastatin $A_4$ prodrug. Three regions of the tumour image in Figure 2A have been used to illustrate the differences in kinetics before and after administration of the drug. Curves of best fit satisfying equation 2 are (**A**) before combretastatin $A_4$ prodrug treatment and (**B**) 160 min after treatment. The most significant change is the reduction in inflow kinetics after administration of the drug. An increase in $T_u$ was seen for regions which showed a large decrease in initial rate of signal enhancement after combretastatin $A_4$ treatment (for example (m) in Figure 2A) and for regions in which the initial-rate analysis did not show a marked change [regions (n) and (o) in Figure 2A] (see Table 1)

$$S = S_0 e^{-\gamma^2 G^2 \delta^2 (\Delta - \delta/3) D} \quad (3)$$

where $\gamma$ is the gyromagnetic ratio (for $^1$H, $\gamma = 26.75 \times 10^7$ $T^{-1}$ $s^{-1}$), $\delta$ is the diffusion gradient duration, $\Delta$ is the time between the diffusion gradients, $G$ is the diffusion gradient strength and $D$ is the diffusion coefficient for the observed species (Stejskal and Tanner, 1965). The images were acquired with TE = 37 ms, TR = 1.5 s, $\Delta$ = 14 ms, and $\delta$ = 10 ms, to give $b$ values ranging from $7.1 \times 10^7$ to $1.8 \times 10^9$ rad$^2$ s m$^{-2}$. Apparent diffusion coefficients were obtained on a pixel-by-pixel basis by fitting the variation in signal intensity with gradient strength to equation 3.

### Histology

Animals were killed and the tumours fixed, embedded in wax, and sectioned (4 μm) to correspond to the MRI plane. The sections were then stained with haemotoxylin and eosin. The difference in thickness of the histological section and MR image plane (2 mm) means that the correspondence between the two cannot be exact.

### RESULTS

Sarcoma F tumours of the size used in this study (approximately 300 mm$^3$) have central regions that give rise to low signal intensity in moderately $T_2$-weighted spin-echo images (Figure 1A, TR = 130 ms, TE = 21.5 ms, acquired in 23 s). These regions corresponded to necrosis observed in histological sections (Figure 1C). After treatment with combretastatin $A_4$ prodrug the area of these regions of low signal intensity increased slightly (Figure 1B). Haemorrhage (observed in histological sections) appeared to give rise to this expansion of low signal intensity around necrotic regions (Figure 1C).

A significant change in the pattern of tumour perfusion after drug treatment was seen in the initial-rate analysis of GdDTPA uptake (Figure 2). The rate of perfusion of the tumour was reduced in part (Figure 2B–D) or all (Figure 2A) of the tumour centre (greater than *c.* 1.5 mm from the tumour edge) in the imaging plane, 3 h after treatment with the drug. The perfusion of the tumour periphery (within *c.* 1.5 mm of the tumour edge) appeared to increase in extent after drug treatment, as indicated by the increased area of high signal intensity in the tumour periphery in the initial-rate maps (Figure 2).

These changes in perfusion pattern were quantified in representative regions of the tumours using the model of Kennedy et al (1994) (equation 2). Curves of best fit for the paramagnetic contribution to relaxivity ($R_{1p}$) during the GdDTPA inflow experiment are shown in Figure 3A and B (the data were taken from the regions shown in Figure 2A). An increase in the time constant for GdDTPA uptake ($T_u$) was observed in the tumour centre after drug treatment (Figure 3 and Table 1). This was seen in central regions where the initial-rate analysis had indicated a reduction in perfusion [(Figure 2A(m)], and also in central regions which did not exhibit a marked change in initial rate of signal enhancement [(Figure 2A(n)]. An increase in $T_u$ for tumour periphery [Figure 2A(o)] after drug treatment was also seen, but this was not significantly different from control values (Table 1). In the peripheral regions the maximal concentration of GdDTPA increased after combretastatin $A_4$ prodrug treatment (Figure 3B). The calculated values of $T_c$ are not quoted in Table 1 as they were of the order of 2000 s and therefore were not well determined by the inflow measurements, which were made over a period of 600 s.

© Cancer Research Campaign 1998

The apparent diffusion coefficient of tumour water (ADC) was determined at up to 1.8 h after treatment with combretastatin $A_4$ prodrug. The ADC maps (not shown) generated from these data showed no significant changes in the mean ADC of tumour water during this period. The mean tumour ADC ($n = 6$) in the plane observed was $0.7 \pm 0.1 \times 10^{-9}$ m$^2$ s$^{-1}$ before treatment, $0.8 \pm 0.1 \times 10^{-9}$ m$^2$ s$^{-1}$ at 0.8 h, and $0.8 \pm 0.3 \times 10^{-9}$ m$^2$ s$^{-1}$ at 1.8 h after treatment (errors are 1 s.d.). The mean ADCs in necrotic regions, $1.3 \pm 0.2 \times 10^{-9}$ m$^2$ s$^{-1}$, and in viable tissue, $0.7 \pm 0.1 \times 10^{-9}$ m$^2$ s$^{-1}$ were similar to the values found by Maier et al (1997) in model breast tumours.

No changes in the levels of NMR-visible phosphorus metabolites in muscle tissue were observed during the 2.5 h after administration of the drug (Figure 4B). However, localized spectra from the tumour indicated a decline in cellular energy status. The signals due to α-, β- and γ-phosphates of NTP in the tumour decreased in intensity (Figure 4A), whereas the signal due to $P_i$ increased in intensity. The phosphomonoester peak, which in many tumours is composed mainly of the resonances from phosphocholine and phosphoethanolamine (Bhujwalla et al, 1994), exhibited no significant change in intensity. The ratio of $P_i$/NTP in these ISIS-localized spectra rose from $0.4 \pm 0.1$ to $0.8 \pm 0.1$ ($n = 6$, errors are 1 s.d.) during the 2.5 h after administration of combretastatin $A_4$ prodrug (Figure 4C). The pH in the tumour decreased from $7.0 \pm 0.1$ before treatment to a minimum of $6.82 \pm 0.03$ ($n = 6$) after 30 min (errors are 1 s.d.) (Figure 4D). After this time the response of the tumours was variable. This was reflected in an increase in the standard deviation of mean tumour pH, which had a value of $6.9 \pm 0.2$ at 150 min.

Control experiments ($n = 6$) indicated that vertical positioning of the animal in the magnet for up to 5 h had no effect on tumour perfusion, as determined from GdDTPA inflow measurements (see Table 1), or from the pH or levels of NMR-visible phosphorus-containing metabolites in the tumours (Figure 4C and D) or underlying muscle tissue (data not shown).

### DISCUSSION

Combretastatin $A_4$ and its soluble phosphate prodrug have been shown previously to cause rapid and selective disruption of tumour vasculature (Chaplin et al, 1996; Dark et al, 1997). The results of the vascular disruption induced by the drug are shown here to be evident in MRI and MRS experiments on an implanted murine tumour model.

The low signal intensity in moderately $T_2$-weighted spin-echo images of the tumours (Figure 1A) correlated with regions of necrosis seen in histological sections of tumours, which contained extravasated erythrocytes (data not shown). After treatment with the drug the regions of low signal intensity expanded slightly (Figure 1B) and this expansion correlated with haemorrhage observed in histological sections taken at the end of the MRI experiment (Figure 1C). The low signal intensity, which was also observed in the images acquired with a longer relaxation delay (TR = 3 s), is attributed to the presence of paramagnetic deoxygenated haemoglobin in these regions (Brooks and Di Chiro, 1987). This enhances the spin–spin ($T_2$) relaxation of water protons and thus reduces their signal intensity in the spin-echo images.

The perfusion characteristics of the tumour underwent significant changes after drug treatment. An initial-rate analysis of GdDTPA inflow data (Figure 2) demonstrated that the alteration to the perfusion characteristics was not uniform across the tumour. In general, the sarcoma F tumour contains both well-perfused (high signal intensity in Figure 2) and poorly perfused regions (low signal intensity). After treatment with combretastatin $A_4$ prodrug, the initial rate of GdDTPA inflow was reduced in some or all of the central regions with relatively high perfusion (Figure 2), whereas the extent of GdDTPA perfusion was increased in peripheral regions of the tumour.

The model of Kennedy et al (1994) was used to analyse regions that had differing characteristics in the initial rate maps. The uptake and clearance of the paramagnetic relaxation agent GdDTPA are described in terms of the time constants for uptake ($T_u$) and clearance ($T_c$), each of which is assumed to follow first-order kinetics. The uptake and clearance of the agent are dependent on the perfusion characteristics of the tissue, and were found to alter after combretastatin $A_4$ treatment.

$T_u$ and $T_c$ were calculated for specific regions of the tumours before and 160 min after administration of combretastatin $A_4$. Before treatment, there was a rapid inflow of the agent into the tissue via the tumour vasculature, followed by a slow clearance resulting from removal of the tracer from the blood stream (Figure 3A). After treatment with the drug the time constant for uptake ($T_u$) was greatly increased (Figure 3B, Table 1). This analysis was more sensitive to reductions in tracer inflow than the initial rate analysis and showed that $T_u$ was increased in regions of the tumour that did not exhibit a significant reduction in the initial rate of contrast agent uptake, as illustrated in Figure 3B. These data showed that drug-induced damage to the vasculature resulted in a significant decrease in the rate of perfusion of the tumour centre (Table 1). The analysis also confirmed the apparent increase in extent of perfusion of the tumour periphery, which was observed in the initial rate maps after drug treatment. Figure 3 shows that the concentration of the contrast agent was increased in the tumour periphery after treatment with the drug, implying that disruption of the vasculature at the centre of the tumour had diverted blood flow to the periphery. This phenomenon of increased peripheral perfusion may be exploitable in enhancing the delivery of a co-administered chemotherapeutic agent targeted at viable tumour cells in peripheral regions of the tumour.

The energy status of the tumour also responded to drug treatment. $^{31}$P-MR spectra were obtained from voxels containing tumour tissue (tumour volume approximately 300 mm$^3$) (Figure 4A) and underlying muscle (1350 mm$^3$) (Figure 4B) for up to 2.7 h

**Table 1** Time constants for uptake ($T_u$) of GdDTPA for tumour centre (pixels more than *c*. 1.5 mm from tumour edge) and periphery (pixels less than *c*. 1.5 mm from tumour edge) in response to treatment with combretastatin $A_4$ prodrug

| | Tumour centre | | Tumour periphery | |
|---|---|---|---|---|
| ***t* (min)** | **$T_u$(s)** | **Control $T_u$(s)** | **$T_u$(s)** | **control $T_u$(s)** |
| 0 | 39 ± 5 | 26 ± 9 | 40 ± 20 | 30 ± 10 |
| 160 | 100 ± 40 | 30 ± 10 | 70 ± 20 | 50 ± 30 |

Data were fitted to equation 2 for experiments at 0 min and 160 min after treatment with combretastatin $A_4$ prodrug (no treatment in the case of controls). The data are means ± 1 s.d. for tumours from $n = 6$ combretastatin-treated animals and $n = 6$ control animals. $T_u$ in tumour centre after combretastatin treatment was significantly different from pretreatment and control values ($P < 0.05$, Student's *t*-test). $T_u$ in tumour periphery after combretastatin treatment was significantly different from pretreatment value ($P < 0.05$, Student's *t*-test), but was not significantly different from $T_u$ of control tumours at 160 min.

© Cancer Research Campaign 1998

**Figure 4** Changes in levels of $^{31}$P metabolites and pH in response to combretastatin $A_4$ prodrug treatment, determined by localized $^{31}$P-MRS. (**A**) Localized spectra from a representative tumour, (**a**) before treatment and (**b**) 30 min, (**c**) 80 min, and (**d**) 150 min after treatment with the drug. The signals from the α-, β-, and γ-phosphates of nucleoside triphosphates (NTP) decrease, and the signal from inorganic phosphate ($P_i$) increases. PME, phosphate monoesters. (**B**) Spectra from muscle beneath the tumour in (**A**), acquired at the same time. PCr, phosphocreatinine. The spectra have been scaled by a factor of 0.5 relative to those in (**A**). (**C**) Tumour $P_i$/NTP ratio (**a**) after combretastatin $A_4$ prodrug treatment and (**b**) control. In both cases, $n = 6$, and error bars are 1 s.d. (**D**) Response of tumour pH to treatment with combretastatin $A_4$ prodrug. Significant differences between pH in (**a**) treated and (**b**) control tumours are indicated (Student's *t*-test, $^{**}P < 0.01$; $^{*}P < 0.05$; nearest time points were used, $n = 6$ for both treated and control)

*© Cancer Research Campaign 1998*

after administration of the drug. No perturbation in the metabolism of the underlying muscle was observed, but there was a steady decrease in the energetic status of the tumour, as indicated by an increasing $P_i$/NTP ratio (Figure 4C). The $P_i$ in these tumours is assumed to be mainly intracellular (Stubbs et al, 1992). An initial decrease in tumour pH was seen at 30 min after drug treatment in all tumours. The pH response of the tumours after this period varied but, on average, remained more acidic than before treatment (Figure 4D). The decline in cellular energy status is presumably the result of the decreased perfusion observed in the MRI experiments (Tozer and Griffiths, 1992). The decrease in pH was assumed to be due to increased lactate concentrations, resulting from decreased oxygenation of the tumours (Steen, 1989).

Diffusion-weighted MRI has demonstrated potential for providing information on tumour oxygenation (Dunn et al, 1995) and necrosis (Maier et al, 1997). Decreased oxygenation has been shown to decrease the ADC of tumour water (Dunn et al, 1995) and necrosis has been shown to increase it (Maier et al, 1997). However there was no significant change in ADC maps acquired before and for up to 1.8 h after treatment with combretastatin $A_4$ prodrug. This may reflect the fact that there was no change in the cellularity of the tumours in these regions (as shown by histology, Figure 1C), whereas in the necrotic regions of two model breast tumours (Maier et al, 1997), in which the water ADC was increased, there was a decrease in cellularity. The reason why there was no decrease in water ADC, despite the fact that the oxygenation of the tumours must almost certainly have been decreased after drug treatment, is not clear.

In conclusion, combretastatin $A_4$ prodrug caused damage to the vasculature of a murine tumour model that resulted in haemorrhage, reduction in tumour perfusion rates, and decreased tumour energy status that could be detected using MRI and MRS experiments. The magnetic field strength and therefore sensitivity of the MR spectrometer used in this study was greater than those used clinically. Nevertheless we believe these techniques, in particular the GdDTPA inflow experiment, could be used in the clinic to determine the response of human tumours to treatment with this drug.

### ACKNOWLEDGEMENTS

The authors wish to thank Professor GR Pettit for kindly providing combretastatin $A_4$ prodrug. This research was supported with grants from the Cancer Research Campaign.

### REFERENCES

Bhujwalla ZM, Shungu DC, He Q, Wehrle JP and Glickson JD (1994) MR studies of tumours: relationship between blood flow, metabolism, and physiology. In *NMR in Physiology and Biomedicine*, Gillies RJ (ed), pp. 311–328. Academic Press: San Diego

Brooks RA and Di Chiro G (1987) Magnetic resonance imaging of stationary blood: a review. *Med Phys* **14**: 903–913

Chaplin DJ, Pettit GR, Parkins CS and Hill SA (1996) Antivascular approaches to solid tumour therapy: Evaluation of tubulin binding agents. *Br J Cancer* **74**: S86–S88

Dark GG, Hill SA, Prise VE, Tozer GM, Pettit GR and Chaplin DJ (1997) Combretastatin A-4, an agent that displays potent and selective toxicity toward tumor vasculature. *Cancer Res* **57**: 1829–1834

Denekamp J, Hill SA and Hobson B (1983) Vascular occlusion and tumour cell death. *Eur J Cancer Clin Oncol* **19**: 271–275

Dunn JF, Ding S, O'Hara J, Liu KJ, Rhodes E and Weaver JB (1995) The apparent diffusion coefficient measured by MRI correlates with $pO_2$ in a RIF-1 tumor. *Magn Reson Med* **34**: 515–519

Gillies RJ, Scherer PG, Raghunand N, Okerlund LS, Martinez-Zaguilan R, Hesterberg L and Dale BD (1991) Iteration of hybridoma growth and productivity in hollow fiber bioreactors using $^{31}P$ NMR. *Magn Reson Med* **18**: 181–192

Hoult DI, Busby SJW, Gadian DG, Radda GK, Richards RE and Seeley PJ (1974) Observation of tissue metabolites using $^{31}P$ nuclear magnetic resonance. *Nature* **252**: 285–287

Kennedy SD, Szczepaniak LS, Gibson SL, Hilf R, Foster TH and Bryant RG (1994) Quantitative MRI of Gd-DTPA uptake in tumours: response to photodynamic therapy. *Magn Reson Med* **31**: 292–301

Latour LL, Svoboda K, Mitra PP and Sotak CH (1994) Time-dependent diffusion of water in a biological model system. *Proc Natl Acad Sci USA* **91**: 1229–1233

Maier CF, Paran Y, Bendel P, Rutt BK and Degani H (1997) Quantitative diffusion imaging in implanted human breast tumors. *Magn Reson Med* **37**: 576–581

Negendank W (1992) Studies of human tumours by MRS: A review. *NMR Biomed* **5**: 303–324

Ordidge RJ, Connelly A and Lohman JAB (1986) Image-selected *in vivo* spectroscopy (ISIS). A new technique for spatially selective NMR spectroscopy. *J Magn Reson* **66**: 283–294

Pettit GR, Singh SB, Hamel E, Lin CM, Alberts DS and Garcia-Kendall D (1989) Isolation and structure of the strong cell growth and tubulin inhibitor combretastatin A-4. *Experientia* **45**: 115–211

Pettit GR, Temple C, Narayanan VL, Varma R, Simpson MJ, Boyd MR, Rener GA and Bansal N (1995) Antineoplastic agents 322. Synthesis of combretastatin A-4 prodrugs. *Anticancer Drug Design* **10**: 299–309

Steen RG (1989) Response of solid tumors to chemotherapy monitored by *in vivo* $^{31}P$ nuclear magnetic resonance spectroscopy: A review. *NMR Biomed* **49**: 4075–4085

Stejskal EO, Tanner JE (1965) Spin diffusion measurements: spin echoes in the presence of a time-dependent field gradient. *J Chem Phys* **42**: 288–292

Stubbs M, Bhujwalla ZM, Tozer GM, Rodrigues LM, Maxwell RJ, Morgan R, Howe FA and Griffiths JR (1992) An assessment of $^{31}P$ MRS as a method of measuring pH in rat tumours. *NMR Biomed* **5**: 351–359

Taylor DJ, Bore PJ, Styles P, Gadian DG and Radda GK (1983) Bioenergetics of intact human muscle. A $^{31}P$ nuclear magnetic resonance study. *Mol Biol Med* **1**: 77–94

Tozer GM and Griffiths JR (1992) The contribution made by cell death and oxygenation to $^{31}P$ MRS observations of tumour energy metabolism. *NMR Biomed* **5**: 279–289

© Cancer Research Campaign 1998