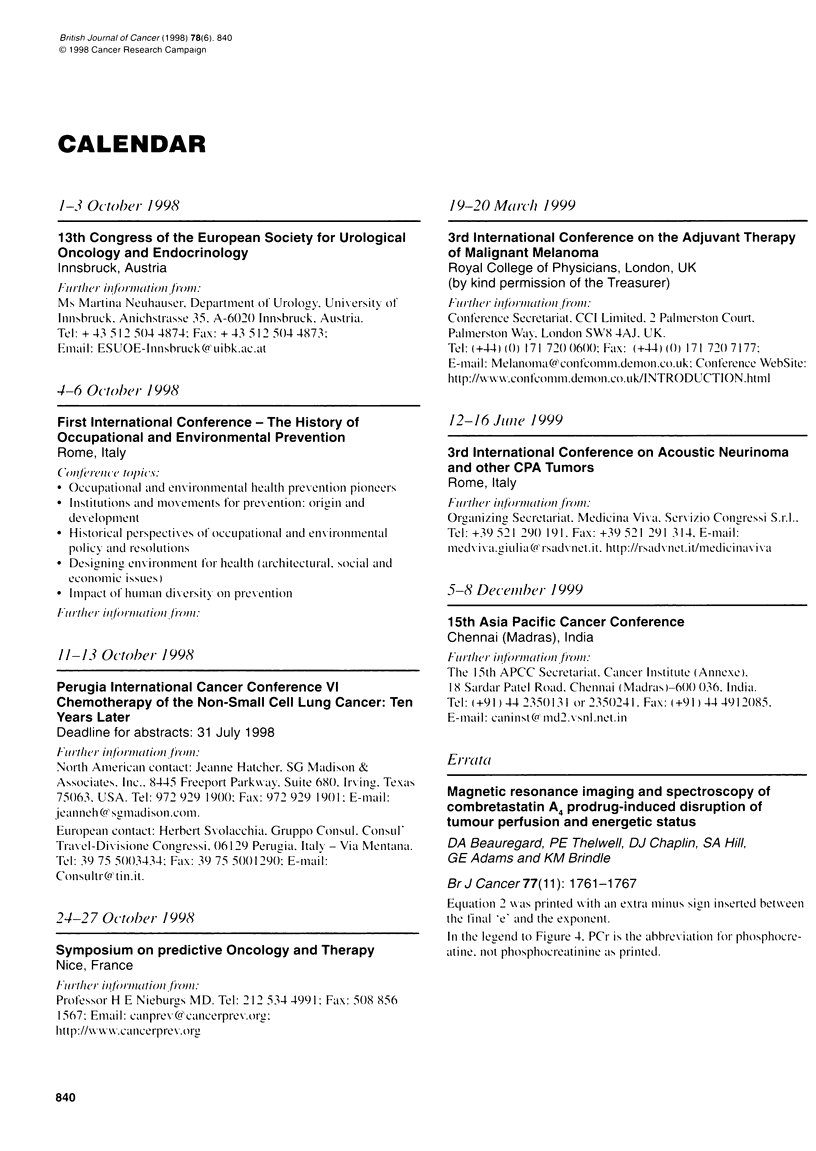# Magnetic resonance imaging and spectroscopy of combretastatin A_4_ prodrug-induced disruption of tumour perfusion and energetic status

**Published:** 1998-09

**Authors:** 


### *Errata*

**Magnetic resonance imaging and spectroscopy of combretastatin $A_4$ prodrug-induced disruption of tumour perfusion and energetic status**

*DA Beauregard, PE Thelwell, DJ Chaplin, SA Hill, GE Adams and KM Brindle*

*Br J Cancer* **77**(11): 1761–1767

Equation 2 was printed with an extra minus sign inserted between the final 'e' and the exponent.

In the legend to Figure 4, PCr is the abbreviation for phosphocreatine, not phosphocreatinine as printed.